# ZnO-Hydroxyapatite-Coated Ti-6Al-4V With Curcumin and Ginger Extract for Load-Bearing Implants

**DOI:** 10.1111/jace.70532

**Published:** 2026-02-11

**Authors:** Arjak Bhattacharjee, Ujjayan Majumdar, William S. Dernell, Amit Bandyopadhyay, Susmita Bose

**Affiliations:** W. M. Keck Biomedical Materials Research Laboratory, School of Mechanical and Materials Engineering, Washington State University, Pullman, Washington, USA

**Keywords:** curcumin, hydroxyapatite, implants, osseointegration, titanium

## Abstract

There is a rising trend in the number of clinical cases related to bone implantation and bone cancer across the globe due to longer life expectancy, accidents, and sports-related injuries. In this study, we have used curcumin and ginger extract as alternate osseointegrating agents with in vitro osteosarcoma inhibition potential for load-bearing, site-specific delivery after direct incorporation on the plasma-sprayed ZnO-doped hydroxyapatite (HA)-coated Ti-6Al-4V. Successful parametric optimization during coating can lead to thickness of 80–150 μm. The in vivo efficacy of this novel localized delivery vehicle for initial-stage osseointegration is tested with a rat distal femur model. Various histological evaluations with Masson–Goldner, hematoxylin and eosin (H&E), and Sanderson rapid bone staining (SRBS) indicate up to ~2 times greater new bone formation surrounding the drug-loaded implants than the control group. The in vitro results indicate that curcumin and ginger extract reduce MG-63 osteosarcoma cell viability on the implant surface by 11-fold. The assessment of antibacterial properties against *Staphylococcus aureus* (S. aureus) shows ~92% efficacy of the treatment samples. After osteosarcoma surgery, these localized drug delivery vehicles can find applications in load-bearing defect repair sites.

## 1 ∣ Introduction

Bone-related clinical conditions led to over a million hip and knee replacement surgeries in the years 2009–2011 and posed ~$215 billion in expenses [1]. One recent study predicts that the osteoporosis-related fracture cases are expected to rise to ~13.6 million in 2030, as compared to ~10.2 million in 2010 [2, 3]. When the defect size in bone exceeds a critical size, surgical implantation with external support is required to ensure sufficient bone growth [4]. Titanium and its alloys are used to reconstruct load-bearing skeletal defects, such as in knee and hip replacements, due to their non-toxicity to the human body, superior corrosion resistance, and high strength-to-weight ratio [5]. This limitation can be addressed by coating Ti with bioactive hydroxyapatite [HA, Ca_10_(PO_4_)_6_(OH)_2_] ceramics [6]. The chemical and structural similarities between HA and human hard tissues facilitate new bone formation after in vivo implantation [7]. The unique crystal chemistry of HA permits the incorporation of several transition-metal cations into the Ca2+ sites, further improving its biological performance [8]. Among these transition metals, Zn^2+^ is an essential trace element that promotes DNA synthesis and new bone formation [9, 10]. Currently, plasma spraying is the only commercially viable manufacturing technique approved by the US Food and Drug Administration (FDA) for producing cera-scoated metallic implants [11]. Plasma-sprayed HA-coated titanium implants demonstrate variable in vivo failure rates depending on anatomical application and follow-up duration [12, 13]. Dental implants show relatively favorable outcomes, with 1.5%–6.8% failure rates and 93.2%–98.5% survival at 4–8 years, comparable to uncoated titanium [14]. However, hip applications reveal concerning patterns. HA-coated acetabular cups exhibit a 70% higher revision risk than uncoated alternatives, with higher aseptic loosening rates [15]. Femoral stem performance varies dramatically by design, ranging from acceptable 2% failure rates to problematic 23% revision rates at 17 years [16, 17]. Knee prostheses demonstrate excellent outcomes with 97.1% survival at 20 years [18]. Critical failure mechanisms include third-body wear from HA particles, coating delamination, and progress, which compromise long-term fixation [19, 20]. Registry data indicate that while HA coating may provide initial biological advantages, these benefits often diminish over time, with some applications showing increased rather than decreased failure risks compared to uncoated implants. Early-stage osseointegration on plasma-sprayed implants is also a significant challenge. One additional challenge is bacterial colonization on the implant surface post-surgery, which may lead to implant failure and necessitate revision surgery. The current approach is to treat this with antibiotics, but localized antibiotic delivery to the implant site is difficult. Additionally, antibiotic-resistant bacteria are another important clinical concern [21].

Osteosarcoma, a more common form of bone cancer, stands as the most prevalent malignancy among pediatric patients and young adults in the United States [22]. Despite significant improvements in osteosarcoma treatment over the last two decades, current treatment options have failed to increase the life span of refractory osteosarcoma patients beyond 5 years [23]. These challenges fuel the requirement to design an alternate strategy for osteosarcoma treatment with localized in vivo drug delivery vehicles [24]. To address these issues, we aim to utilize alternative natural medicinal compounds along with transition metals in the coated implant. One in five US adults prefers naturopathy over synthetic medicines due to its abundance, prolonged effects, and non-toxicity compared to synthetic drugs [25]. One such natural medicinal compound is curcumin, the active compound of turmeric (*Curcuma longa*) [26]. It has been utilized as a food supplement in Asian cuisine since antiquity. Recent research shows that curcumin has chemopreventive properties against different types of cancer cells [27]. Another important natural medicinal compound is ginger extract, from ginger root (*Zingiber officinale Roscoe*) [28]. It has anti-inflammatory properties, and ginger extract is well known to enhance metabolic rate and digestion [29]. Previous clinical trials have reported that the administration of ginger extract is beneficial for patients with osteoarthritis. It also helps in osteoclast reduction and the improvement of bone density [30]. There is a significant knowledge gap in available literature regarding the possibility of utilizing curcumin and ginger extract co-delivery systems as an alternate early-stage osseointegrating agent for different types of bone disorders. Our work aims to bridge this gap. The major research question posed in this work is whether we can use curcumin–ginger extract co-delivery from zinc oxide (ZnO)-doped hydroxyapatite-coated titanium as a localized drug delivery vehicle to enhance initial-stage in vivo osseointegration and simultaneously show in vitro MG-63 osteosarcoma inhibition and antibacterial efficacy.

The objectives of this study are twofold. First, to utilize ZnO-doped HA-coated Ti for co-delivery of curcumin and ginger extract, followed by assessment of its in vitro performance in terms of osteoblast growth, osteosarcoma inhibition, and antibacterial efficacy. Second, to investigate the early-stage in vivo osseointegration potential of this novel delivery system after surgery in a rat distal femur model. It is hypothesized that the direct incorporation of curcumin and ginger extract with ZnO-doped HA-coated Ti will show enhanced early-stage in vivo osseointegration. The novelty of this work is designing a co-delivery system with curcumin and ginger extract after the direct incorporation on ZnO-doped HA-coated Ti. The efficacy of the designed delivery system is evaluated by in vitro osteoblast and MG-63 osteosarcoma proliferation, and an antibacterial effectiveness study against *Staphylococcus aureus*. The in vivo properties are assessed with rat distal femur model surgery and measurement of new bone formation with staining and histology.

## 2 ∣ Materials and Methods

### 2.1 ∣ Substrate Preparation

First, sieving of the commercial HA powder (NEI, USA) was performed using a 175–212 μm mesh to achieve a particle size in that range. To dope ZnO, 0.25 wt.% of ZnO was mixed with the HA, and the mixture was ball-milled for 2 h (powder-to-ball-ratio 2:1). Radio frequency (RF) induction plasma spraying (Tekna Plasma System, Canada) was utilized to coat the undoped and doped HA powders on sandblasted Ti-6Al-4V (Ti64) discs and cylinders. Parametric optimization was carried out as per our previous works to ensure sufficient coating strength [7, 11]. The utilized parameters were 25 kW power, 110 mm spray distance, and 25 standard L min^−1^ (s.L.p.m.) of carrier gas flow rate. The amplitude was fixed in the range of 20–30, and a frequency of 106.8–107.3 Hz was used during the coating preparation. Further details about parametric optimization and resultant coating thickness are mentioned in Table 1.

### 2.2 ∣ Phase Identification and Evaluation of Mechanical Properties

x-Ray diffraction method (Siemens D500 diffractometer) was used to identify the phases present in the coating at a 2*θ* range of 20–50 degrees, with Cu K*α* radiation at 35 kV and 30 mA. To assess the bond strength test of the fabricated coatings, the ASTM C633 standard method was utilized [31, 32]. According to this method, a coating thickness of ~285 ± 20 μm was used during tensile testing. Armstrong A-12 epoxy resin was used to glue each sample to the posts, followed by curing at 93.3°C (200°F) for 30 min and cooling down to room temperature. The tensile test was performed at a constant crosshead speed of 0.0013 cm s^−1^ until the failure of the coating. The adhesion strength was computed using the failure load/sample area data.

### 2.3 ∣ Drug Loading and Release Studies

In vitro release of curcumin and ginger extracts was measured in phosphate-buffered saline for over 28 days. Curcumin was purchased from Millipore Sigma, and ginger extract was obtained using the Soxhlet extraction method from ginger root, as described in our previous work [29]. The drugs were sterilized by passing through a 0.22 μm filter and drop-casted on top of the coated surface (500 μg of curcumin, 100 μg of ginger extract), and the drug-loaded sample was put inside a glass vial, filled with 4 mL of PBS. The vials were placed in a shaker (150 rpm) at 37°C. The solutions were replaced with fresh PBS after collecting them at each time point. Curcumin and ginger extract release were quantified by measuring 100 μL of the drug solution at 427 nm and 281 nm respectively, using a Biotek (VT, USA) Synergy 2 SLFPTAD microplate reader. The release of curcumin and ginger extract was calculated using a standard calibration curve obtained from their release at varying concentrations. Weibull model (using MATLAB 2021b) was employed to analyze the release kinetics data, as per the following equation.


(1) $$Z(t)=100\times \left\{1-\text{exp}\left[-\frac{t^{r}}{s}\right]\right\}$$

In the provided equation, the drug release (%) is Z(t), and t is the time in days. r denotes the shape parameter, and s denotes the scale parameter.

### 2.4 ∣ In Vitro Cell-Material Interaction

In vitro properties of the designed drug delivery system were characterized by osteoblast and osteosarcoma cell cultures.

#### 2.4.1 ∣ Osteoblast Cell Culture

Human fetal osteoblast cells (hFOB 1.19, obtained from ATCC, Manassas, VA, USA) were cultured on the fabricated samples for 3, 7, and 11 days [33]. The cell culture media was made using a 1:1 mixture of Dulbecco’s modified Eagle’s medium and Ham’s F12 medium (DMEM/F12, Sigma, St. Louis, MO, USA). The supplemental media was made with 2.5 mM sterilized L-glutamine solution along with fetal bovine serum (10% FBS, ATCC, Manassas, VA, USA) and 0.3 mg/mL G418 (Sigma, St. Louis, MO, USA). To ensure the sterility of the media, 0.1% penicillin–streptomycin was used. First, the samples after sterilization were put on a 24-well plate, and 40 × 10^3^ cells were loaded on each sample. Next, 1 mL of cell culture media was added to each well, and the plates were incubated at 34°C in a 5% CO_2_ atmosphere.

The viable cell count was quantified with an MTT assay [3-(4,5-dimethylthiazol-2-yl)-2,5-diphenyltetrazolium bromide]. During the assay, the old media were removed, and 100 μL of MTT solution was added to each sample. After that, each well was filled with 900 μL of media. The plates were then put inside an incubator at 34°C for 1 h. After 1 h, the MTT solution was removed and each well was filled with 600 μL of MTT solubilizer (10% Triton X-100, 0.1 N HCl, and isopropanol) to dissolve the purple formazan crystals. After this, 100 μL of the dissolved crystal solution was put into a 96-well plate, and the optical density was quantified at 570 nm with a UV–VIS microplate reader.

To investigate the cellular morphology, FESEM was used [34]. After each time point, the samples were fixed with 2% paraformaldehyde and 2% glutaraldehyde in 0.1 M phosphate buffer and refrigerated overnight at 4°C. After this, the samples were rinsed with 0.1 M phosphate buffer solution (3 times), and post-fixation was performed with 2% osmium tetroxide (OsO_4_). Dehydration was carried out in an ethanolic series from 30% to 90% ethanol once and three times with 100% ethanol. After this, the samples were submerged in hexamethyldisilane (HMDS) and kept overnight inside a desiccator for drying. Before taking the FESEM images, the samples were gold-coated.

#### 2.4.2 ∣ Osteosarcoma Cell Culture

The prepared samples were cultured with the human osteosarcoma cell line (MG-63, ATCC, USA) to assess their in vitro chemopreventive potential. The cell culture was conducted in Eagle’s Minimum Essential Medium and 10 % inactivated FBS (EMEM, ATCC, USA), at a cell density of 35,000-40,000cells in each sample. The cellular viability and attachment on the sample surface were investigated with an MTT assay and FESEM, respectively, using a protocol similar to that of the osteoblasts.

### 2.5 ∣ Measurement of Antibacterial Efficiency

The fabricated samples were tested against *S. aureus* to assess their antibacterial potential as per the modified ISO 22196: 2011 Standard [35]. First, the bacterial activation was performed as per the protocols. After this, the optical density of the activated bacteria was quantified at 625 nm as per the McFarland standard. In the next step, sterilized samples were placed in 24-well plates, and the samples were loaded with 10^6^ CFU of activated bacteria, followed by 1 mL of broth media added per well. After this, the incubation of the well plates was done at 37°C and 90% humidity. After the sample–bacterial interaction, samples were transferred to a glass vial and submerged in 1 mL PBS and vortexed for 15 s to take the attached bacteria out from the sample surface. After this, 10 μL of the diluted bacteria solution was dropwise added to each agar plate, and the plates were kept at 37°C for 24 h inside an incubator. The bacterial colony numbers were counted from the agar plate photographs. Quantification of the bacterial cells was carried out with Equation (2). The antibacterial efficiency of the treatment samples was calculated as 100 – bacterial cell viability (%).


(2) $$\text{Bacterial cell viability}\;(\%)=N_{\text{treatment}}∕N_{\text{control}}\times 100\%$$

In Equation (2), $N_{\text{treatment}}$ and $N_{\text{control}}$ are the numbers of bacterial colonies on the treatment and control agar plates, respectively. The antibacterial efficacy is calculated as per our previous work [7, 26].

### 2.6 ∣ Assessment of In Vivo Properties

#### 2.6.1 ∣ Rat Distal Femur Model

The in vivo bone-forming ability of the fabricated samples was tested using a bilateral, unicortical rat distal femur model, following an Institutional Animal Care and Use Committee (IACUC) protocol approved by Washington State University, Pullman, WA, USA. The surgery was performed in Sprague-Dawley (Envigo, Wilmington, MA, USA) rats weighing 320–340 g. After surgery, the rats went through post-op care and were monitored for up to 6 weeks. Euthanization of the rats was done in Week 6. Before surgery, each rat was placed in a single cage and kept in a room on a 12 h light-dark cycle, with prescribed temperature and humidity. Anesthesia was induced and maintained with IsoFlo (Isoflurane, USP, Abbott Laboratories, North Chicago, IL, USA) and oxygen. The defects were produced using a stepwise increase in drill size to 2.5 mm. Implants with a 2.5 mm diameter and 4 mm height were then placed in the defect site. After the implantation, suturing and stapling were done to close the incision. During the post-op care, the rats were administered subcutaneous meloxicam for 3 days to minimize pain after surgery. Euthanasia was performed through an overdose of CO_2_. Femur samples were harvested after euthanasia and placed in a 10% neutral-buffered formalin solution.

#### 2.6.2 ∣ Histomorphology and Histochemical Analysis

After keeping the harvested femurs in ethanol for 72 h, ethanolic series dehydration was performed, and samples were rinsed with acetone. The dehydrated samples were embedded in methylmethacrylate (iMMA). After this, cross-sections were made perpendicular to the implant axis using a diamond saw. In the next step, the obtained tissue sections were glued to glass slides and then ground and polished. The samples were polished up to a thickness of ~10 μm and stained with Sanderson’s rapid bone staining (SRBS) [Sanderson’s RBS + van Gieson (S-CP3), Dorn and Hart, Microedge Inc., Loxley, AL, USA], Hematoxylin [Sigma Aldrich (H3136), St. Louis, MO, USA] & Eosin [Surgipath, Leica, Buffalo Grove, IL, USA] (H&E) staining, and Masson–Goldner’s Trichrome staining [Sigma Aldrich, USA] for the quantification of new bone formation surrounding the implant [36-38]. The stained samples were imaged using a high-magnification field optical microscope. The quantification of new bone around the implant-bone interface was carried out with RGB analysis and area quantification by ImageJ software.

## 3 ∣ Results

### 3.1 ∣ Coating Optimization, Phase Identification, and Mechanical Properties Assessment

The plasma spraying process involves many variables that must be precisely controlled to achieve optimal coating properties. Critical parameters, including plasma power (22–28 kW), working distance (90–130 mm), gas flow rates, and powder feed rates, directly influence coating crystallinity, phase purity, thickness, and porosity. Even minor variations in these parameters can result in significant inconsistencies in coating quality and performance [39]. To optimize the coating thickness, different combinations of processing parameters in terms of amplitude and frequency (Hz) are utilized during sample preparation. Table 1 reports the utilized parameters to achieve the desired coating thickness. An amplitude of 20–30 and a frequency of 106.8–107.3 Hz is utilized for coating fabrication. The obtained coating shows a thickness in the range of 80–150 μm depending on the sample. Further details about the coating optimization are presented in our previous work with samples of the same compositions [7]. The XRD results (Figure S1) of HAP and Zn-HAP show no undesirable phase formation because of doping and high-temperature plasma-sprayed coating. The obtained peaks match well with hydroxyapatite (JCPDS # 09–0432) [7]. The adhesion strength results show up to ~19.6 ± 3 MPa strength of the obtained coating. Doping with ZnO does not have any negative influence on the adhesion strength. The obtained strength is within the acceptable limit as suggested by the ISO 13779-2 standard [40].

### 3.2 ∣ Drug Release Study

**FIGURE 1 ∣:**
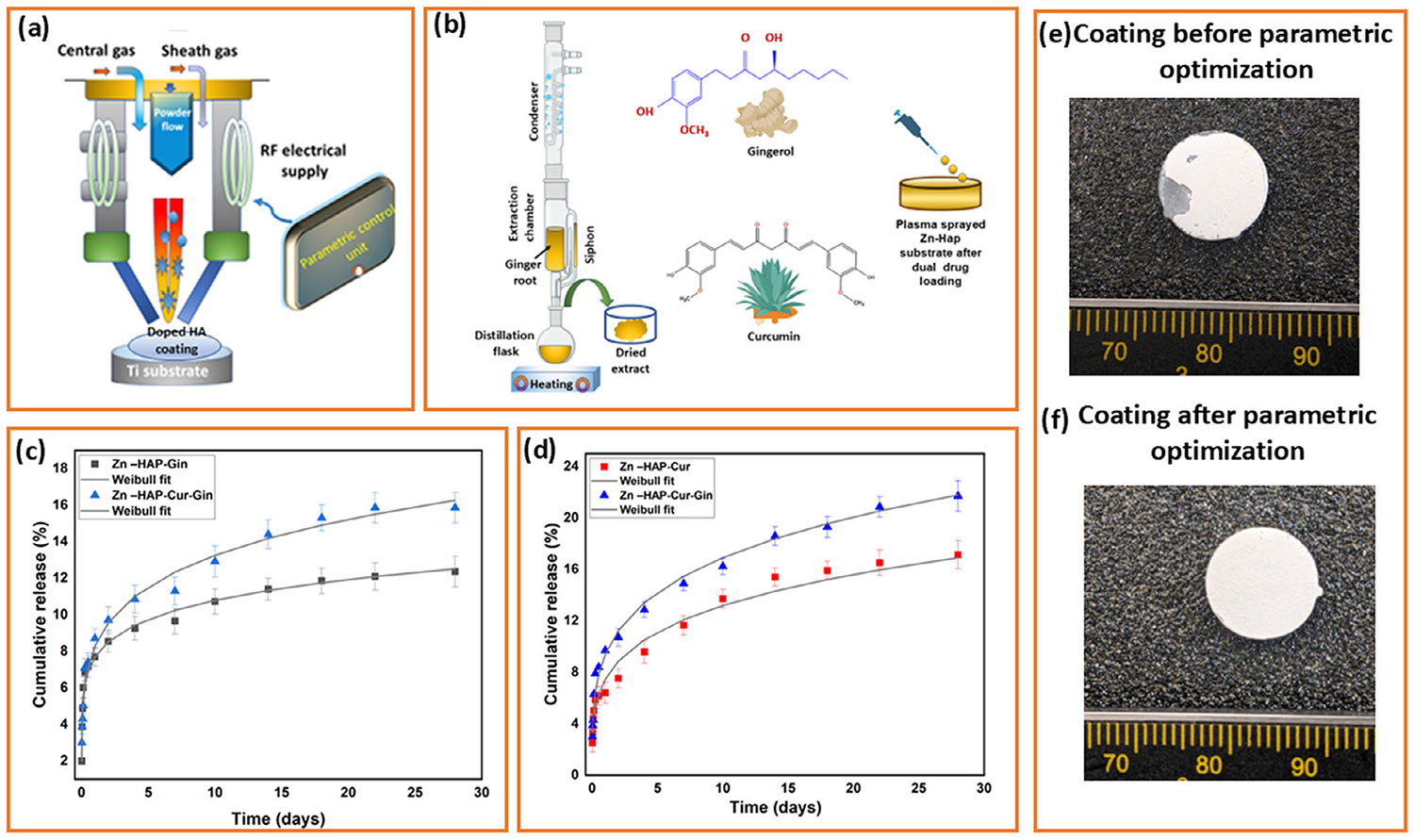
(a) RF induction plasma spraying process to prepare Zn-doped HA and HA-coated Ti, (b) Soxhlet extraction from ginger root. The chemical structure of gingerol, the main component of ginger extract. The chemical structure of another natural medicinal compound, curcumin, is used in this study. Loading of ginger extract and curcumin on Zn-doped HA-coated Ti, (c) cumulative release of ginger extract from Zn-HAP substrate at pH 7.4 shows ~9% release after 28 days. In the presence of curcumin, the release of ginger extract increases to ~15% during the whole period. (d) Cumulative curcumin release from Zn-HAP substrate at pH 7.4 indicates ~12% release after 28 days. In the presence of ginger extract, the release increases to ~20% during the study period, (e) coating before parametric optimization, and (f) coating after parametric optimization.

The cumulative release of ginger extract and curcumin after 28 days from the Zn-HAP substrate at pH 7.4 is shown in Figure 1c,d, respectively. The ginger extract shows ~9% release, and in the presence of curcumin, it increases to ~15% from the Zn-HAP-Cur-Gin substrate. Cumulative curcumin release from Zn-HAP substrate at pH 7.4 shows ~12% release after 28 days. In the presence of ginger extract, it increases to ~20% during the whole study period. Figure 1e,f shows coated samples before and after parametric optimization, respectively.

### 3.3 ∣ In Vitro Cell-Material Interaction and Antibacterial Studies

**FIGURE 2 ∣:**
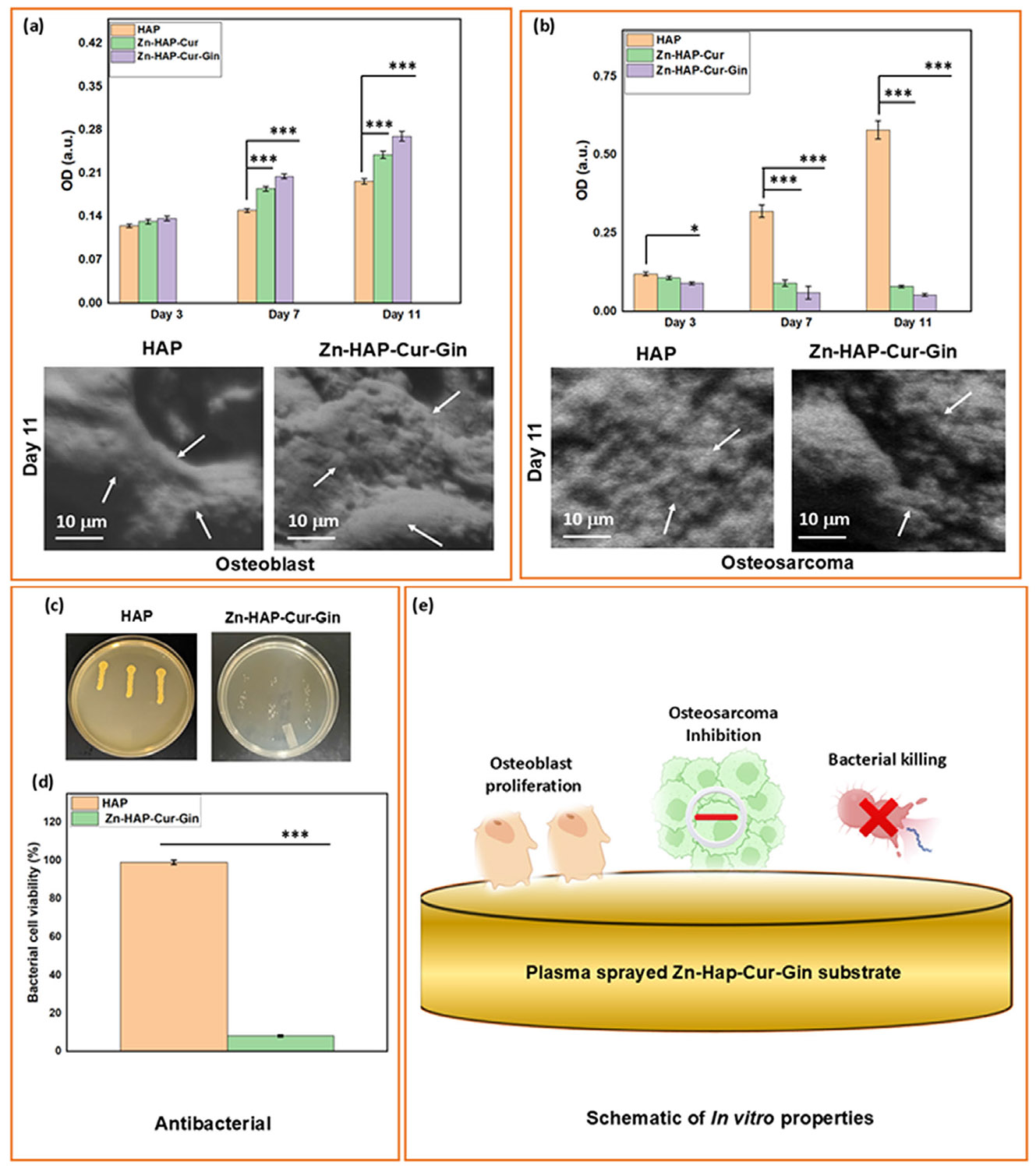
Assessment of in vitro properties of the drug-loaded coated implants (a) MTT assay indicates that no compositions are cytotoxic. On Day 3, the tested compositions show similar cell viability. Significantly higher viability of cells is noticed on the treatment on Days 7 and 11 as compared to the control. The Zn-HAP-Cur sample shows ~1.3 times enhanced cell viability, and ~1.5 times enhancement in cell viability is observed for the Zn-HAP-Cur-Gin composition. A *p* value of <0.001 is denoted by ***. The FESEM images on Day 11 show good cellular attachment covered within the apatite layer. Arrows in the FESEM images denote osteoblast cells. (b) MTT assay after osteosarcoma cell–implant interactions indicates that the cell viability significantly decreases due to curcumin and ginger extract. On Day 3, the Zn-HAP-Cur composition shows a ~1.2-fold reduction in cell viability compared to the control. On Days 7 and 11, further reduction in cellular viability is noticed, with a ~3.7 times reduction on Day 7 and a ~7.3 times reduction on Day 11. The Zn-HAP-Cur-Gin composition shows ~11 times decrease in osteosarcoma viability on Day 11. The FESEM images show the presence of cells and are marked with arrows, (c) the agar plate photographs after implant–*S. aureus* interaction for 36 h show a significant reduction of bacteria on the treatment sample as compared to the control, (d) quantification of bacterial cell viability indicates that The Zn-HAP-Cur-Gin composition shows ~92% antibacterial efficacy *** denotes a *p* value of < 0.001, (e) schematic of the osteoblast proliferation, osteosarcoma inhibition, and bacterial killing due to the actions of Zn^2+^, curcumin, and ginger extracts.

The MTT assay results (Figure 2a) after interactions of osteoblasts with the coated implants indicate that no compositions are cytotoxic. Similar cell viability of all compositions is noticed on Day 3. On Days 7 and 11, the treatment compositions showed higher cellular viability as compared to the control sample. The Zn-HAP-Cur shows ~1.3 times enhanced cell viability, and ~1.5 times enhancement in cell viability is found for the Zn-HAP-Cur-Gin composition. The FESEM images show good cellular attachments covered within the apatite layer. The osteoblast cells are shown with arrows.

The MTT assay quantification after osteosarcoma cell–implant interactions (Figure 2b) indicates a significantly reduced cell viability in the presence of curcumin and ginger extract. The Zn-HAP-Cur composition shows a ~1.2 times decrease in cell viability as compared to the control on Day 3. On Days 7 and 11, it leads to a further reduction in cellular viability, with a reduction of ~3.7 times on Day 7, which leads to ~7.3 times on Day 11. The Zn-HAP-Cur-Gin composition shows ~11 times decrease in cell viability on Day 11. An arrow marks the observed cells on the sample surfaces in the FESEM images.

The antibacterial assay results after implant–*S. aureus* interaction for 36 h are shown in Figure 2c,d. The agar plate photographs (Figure 2c) show a significant decrease in bacterial colonies on the treatment composition compared to the control. Agar plate colony count results (Figure 2d) indicate that the Zn-HAP-Cur-Gin composition shows ~92% antibacterial efficacy. Figure 2e schematically shows the antibacterial efficiency, osteoblast proliferation, and osteosarcoma inhibition on the sample surface.

### 3.4 ∣ Assessment of In Vivo Properties

**FIGURE 3 ∣:**
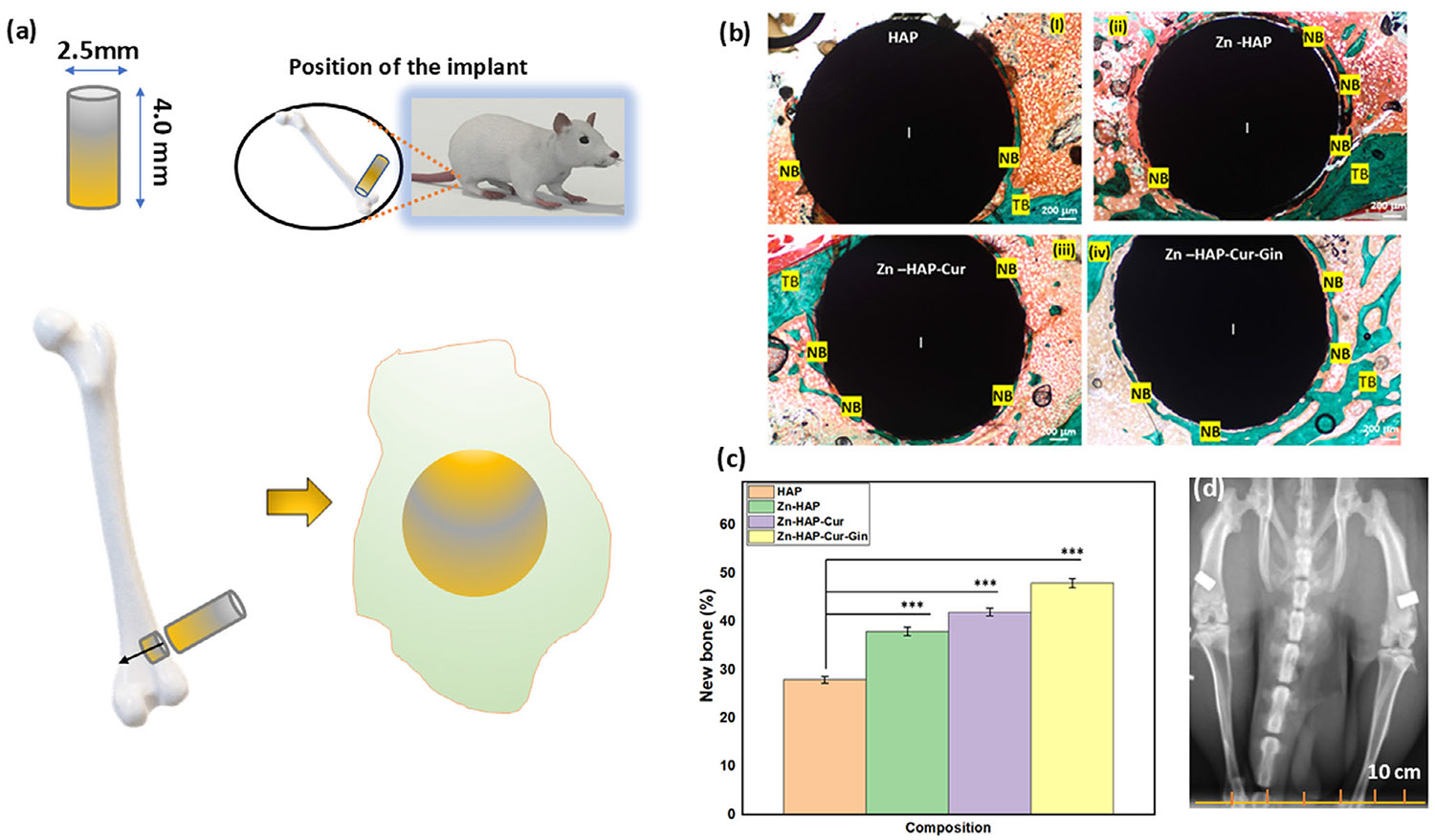
Schematic of in vivo rat distal femur model surgery, Masson–Goldner staining, new bone formation quantification, and radiograph of implant location after rat sacrifice. (a) Experimental design is schematically shown with the drug-loaded implants followed by surgery in a rat distal femur model and cutting of the embedded samples, (b) optical microscopic images after Masson–Goldner staining. In these images, “I” indicates the implant area, “NB” denotes the new bone formation, and “TB” represents the trabecular bone area. The new bone formation is stained green in these images. (c) New bone formation quantification shows ~1.4 times increased bone formation in the Zn-HAP sample, which increases to ~2 times in the Zn-HAP-Cur-Gin composition. (d) A rat radiograph after 6 weeks of surgery shows no presence of fracture and correct implant location.

**FIGURE 4 ∣:**
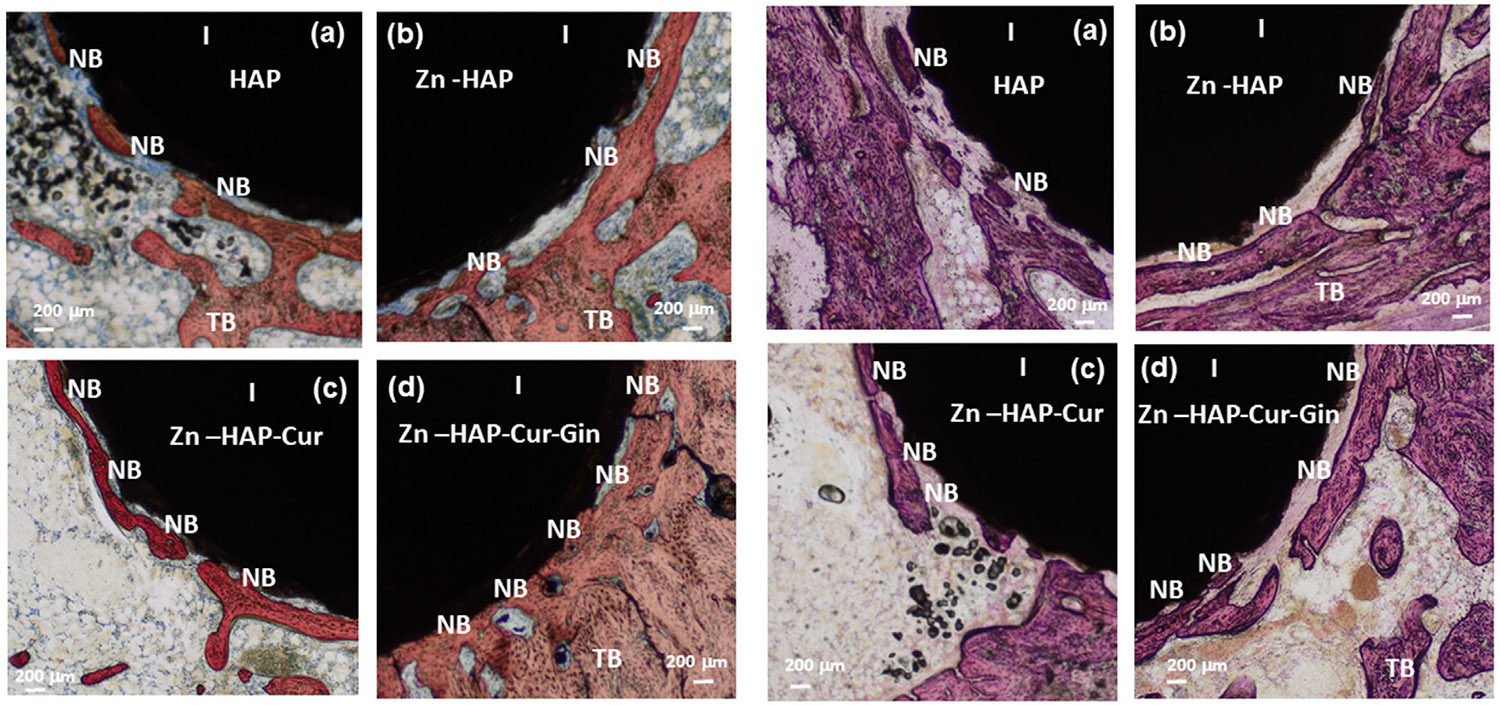
Optical microscopic images after SRBS and H&E staining are shown in (a)–(d) and (e)–(h), respectively. In these figures, “I” denotes the implant area, “NB” denotes the new bone formation surrounding the implant, and “TB” denotes the trabecular bone. The SRBS stained the new bone as light red, and the H&E images stained the new bone pink.

**FIGURE 5 ∣:**
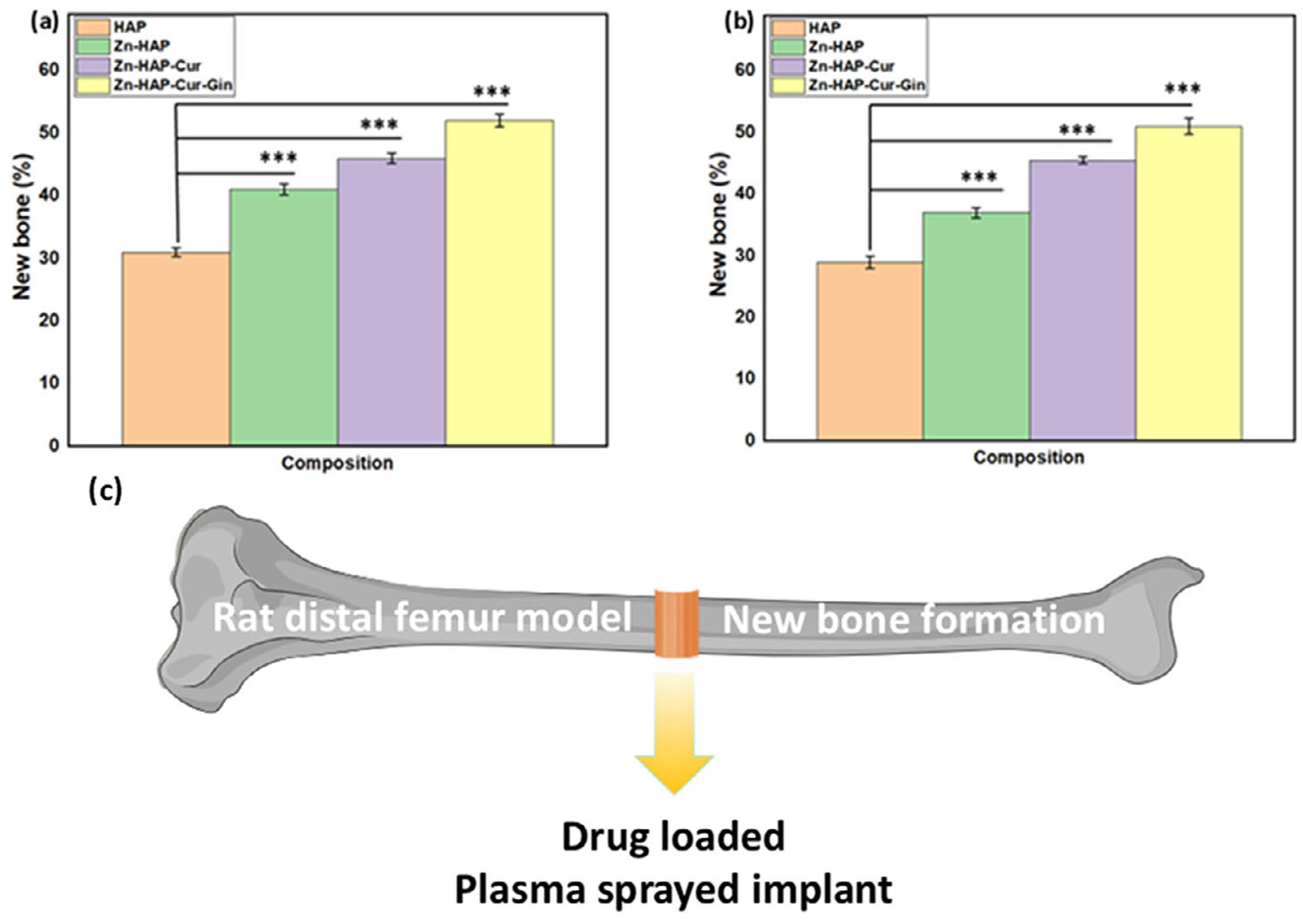
The results after quantification of SRBS and H&E-stained cross sections (a) The SRBS-stained cross sections show ~1.9 times new bone formation surrounding the implant for the Zn-HAP-Cur-Gin composition. The Zn-HAP and Zn-HAP-Cur compositions show ~1.3 times and ~1.4 times enhancement in bone formation, respectively. (b) The H&E-stained cross sections show ~2 times new bone formation for the Zn-HAP-Cur-Gin composition. The Zn-HAP and Zn-HAP-Cur compositions show ~1.6 times and ~1.7 times new bone formations, respectively. *** denotes a *p* value of < 0.001, (c) Schematic of the rat distal femur bone after implantation and new bone formation surrounding the implant.

The in vivo rat distal femur model surgery schematic, Masson–Goldner staining, new bone formation, and radiographs of implant location after rat sacrifice are depicted in Figure 3. Figure 3a shows the in vivo experimental design and implantation of the drug-loaded implants, followed by cutting and staining of the embedded samples. The optical microscopic images of the Masson–Goldner staining are shown in Figure 3b. In these images, “I” indicates the implant area, “NB” denotes the new bone, and “TB” represents the trabecular bone area. The new bone formation is stained green in these images [36]. Measurement of new bone formation (Figure 3c) shows ~1.4 times increased bone formation in the Zn-HAP sample and ~2 times higher new bone formation in the Zn-HAP-Cur-Gin composition, respectively. The rat radiographs after 6 weeks of surgery (Figure 3d) show no presence of fracture and confirm correct implant location. The optical microscopic images after SRBS and H&E staining are shown in Figure 4. The implant area is denoted by “I,” and “NB” indicates the new bone formation surrounding the implant. The trabecular bone area is denoted by “TB.” The new bones are stained as light red in the SRBS staining (Figure 4a-d), whereas the H&E images (Figure 4e-h) stained those as pink [37, 38]. The results after quantification of SRBS and H&E-stained cross sections are presented in Figure 5a,b, respectively. Quantification of the SRBS-stained cross sections (Figure 5a) indicates ~1.9 times new bone formation surrounding the implant for the Zn-HAP-Cur-Gin composition. The Zn-HAP and Zn-HAP-Cur compositions show ~1.3 times and ~1.4 times enhancement in bone formation, respectively. The H&E-stained cross sections (Figure 5b) show ~2 times more new bone development for the Zn-HAP-Cur-Gin composition, and the Zn-HAP and Zn-HAP-Cur compositions show ~1.6 times and ~1.7 times new bone formation, respectively. The schematic of the rat distal femur bone after implantation and the formation of new bone surrounding the implant is shown in Figure 5c.

## 4 ∣ Discussions

### 4.1 ∣ Clinical Significance of Localized Delivery for Orthopedic Applications

Due to a significant increase in osteoporosis-related fractures in the last two decades, enhancing the lifetime of implants is one of the major goals of current orthopedic biomaterial manufacturing [2, 39]. However, post-surgical bacterial infection on the implant surface may lead to implant failure, often requiring revision surgeries. The current approach to minimize the chances of implant infection is by utilizing antibiotics [26]. Delivery of antibiotics to the specific surgical site poses a significant challenge. Recent research shows that antibiotic resistance is on the rise among patients [21]. This alarming situation necessitates the development of a multifunctional orthopedic implant that can promote new bone formation after surgery, along with inherent antibacterial properties. The design goal of our work is to fabricate Zn-doped HA-coated Ti by plasma spraying, followed by the loading of natural medicinal compounds that can promote new bone formation, along with antibacterial efficacy.

### 4.2 ∣ Doped HA-Coated Ti as a Load-Bearing Implant

HA-coated Ti implants are a clinically relevant choice for various load-bearing applications, such as total hip replacement. Bioactive HA on the Ti implant enhances osseointegration after implantation. Previous works report that doping HA with transition metal cations, such as Zn^2+,^ helps to further improve its biological properties and new bone formation [32]. The ionic radius of Zn^2+^ is ~0.74 Å, and as a cationic dopant, it replaces the Ca2+ sites of HA. Zn^2+^ doping significantly contributes to antibacterial properties and osteoblast proliferation. To ensure clinical applications of HA- or doped HA-coated Ti as a load-bearing implant, the ISO 13779-2 standard recommends adhesion strength is ≥15 MPa [40]. Our work shows that a successful coating optimization leads to an adhesion strength of up to ~19.6 ± 3 MPa, which is acceptable as per the FDA recommendations [40].

### 4.3 ∣ Multifunctional Implant Design Strategies

Our strategy of direct incorporation of curcumin and ginger extract with ZnO-doped HA-coated Ti has many unique advantages that lead to the multifunctionality of the implants, such as (a) inherent antibacterial properties, (b) enhanced osteoblast growth on the implant surface, (c) significant inhibition of MG-63 osteosarcoma, and (d) early-stage in vivo osseointegration. Figure 2a shows that in the presence of ZnO, curcumin, and ginger extract, the osteoblast viability significantly increases as compared to the control. Zn^2+^ has the potential to increase the OPG/RANKL ratio, which enhances osteoblast proliferation. Available literature reports suggest that Zn^2+^ triggers the mitogen-activated protein (MAP) kinase pathway and enhances osteoblast cells [41]. Curcumin enhances Runx2 and osteocalcin mRNA expressions, which results in the enhancement of osteoblast cell viability [42]. Curcumin also promotes osteoblast differentiation by upregulating the heme oxygenase 1 (HO-1) pathway [43]. The antibacterial properties of the fabricated drug delivery system are evident from Figure 2c. Zn^2+^ kills bacterial cells through two major mechanisms: (a) by the formation of reactive oxygen species (ROS) after targeting the bacterial cell wall, (b) by selective binding with bacterial DNA and PsaBCA transporters. This selective binding hinders bacterial cells from the uptake of the essential element Mn^2+^. Prevention of Mn^2+^ intake by bacterial cells impairs cell nutrition and leads to their death due to the formation of the Zn^2+^–PsaBCA complex [44]. The polyphenolic groups, present in curcumin and ginger extract, generate ROS and lead to bacterial cell death [45]. Our goal is to fabricate a multifunctional implant that shows antibacterial properties with enhanced osteoblast viability and osteosarcoma inhibition. The selection of ginger extract and curcumin as the natural biomolecules and ZnO as a dopant is useful. Direct incorporation of ginger extract and curcumin on the ZnO-doped HA-coated Ti is utilized as a localized drug delivery vehicle in our work. Drug release from ceramic-coated implants depends on several major factors, including (a) degradation of the matrix, (b) diffusion, (c) electrostatic interaction, and (d) hydrophobic–hydrophilic interactions [46]. The dual interaction of curcumin and ginger extract helps to increase their release in the medium.

### 4.4 ∣ In Vivo Properties Assessment

The in vivo results indicate that curcumin and ginger extract are helpful in new bone formation in the presence of ZnO. The microscopic images (Figures 3 and 4) indicate a higher bone–implant area in the presence of ZnO, curcumin, and ginger extract than that of the control. This is due to the osteogenic potential of these individual elements. Image analysis and quantification reveal that the combined effects of ginger extract, curcumin, and ZnO help in osteoid formation. It further indicates better implant–bone interfacial contact and higher trabecular bone formation surrounding the implant. This can be attributed to the dual effects of these components on both osteoblast enhancement and osteoclast reduction. Ginger extract is reported to enhance collagen type I, alpha I (COL1A1) expression, which in turn promotes osteoblast differentiation and bone marrow cell differentiation [29]. Our work corroborates this hypothesis, as evidenced by the higher bone formation in the presence of curcumin and ginger extract. Previous works report that curcumin immobilization on implant surfaces decreases encapsulation of fibrous tissue and increases bone–implant contact area [11]. The results obtained in our work corroborate these observations.

### 4.5 ∣ Curcumin and Ginger Extract as an Alternate Chemopreventive Agent

The existing treatment options for osteosarcoma involve prolonged drug administration, surgical procedures, and post-operative chemotherapy [22, 23]. This approach presents significant challenges, such as (a) the high cost of the drugs, (b) the toxicity of the drugs to healthy organs, (c) the complex drug schedule, and (d) prolonged hospital stay [11]. To mitigate these challenges, naturally derived biomolecules like curcumin and ginger extract may emerge as safer alternatives. These medicinal compounds are readily available, have sustained positive effects on human health, and exhibit minimal adverse effects on the body [47, 48]. Our strategy is to use curcumin and ginger extract as a combined delivery system with plasma-sprayed ZnO-doped HA-coated Ti implants to repair critical-sized defects after osteosarcoma surgery. Turmeric and ginger are globally recognized as culinary spices, and their medicinal properties are well documented in the literature. They have medicinal properties such as anti-inflammatory effects and immune system enhancement and can be utilized in various bone-related disorders. Recent research suggests that curcumin possesses the potential to influence various cell signaling pathways responsible for malignant tumor development [27]. In this way, curcumin restricts cancer cell growth. Curcumin mainly inhibits nuclear factor-*κβ* (NF-*κβ*) pathways. Ginger extract accelerates cellular apoptosis, down-regulates cyclin D1, and inhibits NF-*κβ* pathways, which leads to cancer cell death [29]. The NF-*κβ* is present as a conjugate with I*κβ* in the cytoplasm of static cells. This conjugate stays as an inactive compound [49]. Phosphorylation of IK*β* is a significant step by which the NF-*κβ* gets activated, transported to the nucleus, and forms chemical bonds with the DNA. This series of events initiates cancer cell propagation. Curcumin and ginger extract have the potential to inhibit phosphorylation, which further decreases NF-*κβ* pathway activation. This leads to the arrest of cancer cell growth and inhibits their propagation. In the current work, the osteosarcoma cell culture quantification (Figure 2b) shows that the presence of curcumin and ginger extract significantly reduces cellular viability. Local drug delivery to the surgery site is difficult, and current methods fail to show significant progress in this regard [50]. We utilized an alternative approach by directly loading the drugs on top of the implants, which is expected to mitigate the challenges associated with localized drug delivery after cancer surgeries.

## 5 ∣ Conclusions

Our research demonstrates that Ti-6Al-4V implants coated with ZnO-doped HA and loaded with curcumin and ginger extract improve in vivo early-stage osseointegration and enhance in vitro performance. The adhesion strength of these coated implants surpasses the minimum recommendation set by the FDA. The major conclusions of this study are:

- Quantification of individual drug release at a physiological pH of 7.4 shows approximately 9% release of ginger extract and approximately 12% release of curcumin, respectively.
- Histomorphology findings reveal up to a 2-fold increase in osseointegration after 6 weeks of surgery in a rat distal femur model, attributed to the combined effects of curcumin and ginger extract.
- In vitro results demonstrate enhanced osteoblast viability and up to ~92% increased antibacterial efficacy.
- A substantial reduction of approximately 11-fold is observed in the MG-63 osteosarcoma cell viability on the surface of drug-loaded implants.
- These multifunctional drug delivery systems may be used for potential applications in load-bearing orthopedic sites.

## Supplementary Material

Supplementary

Additional supporting information can be found online in the Supporting Information section.

## Figures and Tables

**TABLE 1 ∣:** List of coating parameters such as amplitude, frequency (Hz), and the resultant coating thickness.

Sample	Amplitude range	Frequency range (Hz)	Coating thickness (μm)
HAP	20–30	106.8–107.3	90–150
Zn-HAP	20–30	106.8–107.3	80–145

## Data Availability

Data presented in this manuscript will be made available upon reasonable request to the corresponding author.
