# Supplementary material for: ZnO-Hydroxyapatite-Coated Ti-6Al-4V With Curcumin and Ginger Extract for Load-Bearing Implants
Source: J Am Ceram Soc. Author manuscript; Available in PMC 2026 May 20. (PMC13186412; doi:10.1111/jace.70532)
Supplement: Supplementary [file NIHMS2168576-supplement-Supplementary.pdf]

1).

ZnO-Doped Hydroxyapatite-coated Titanium with Curcumin and Ginger Extract for Load-Bearing Implants

Arjak Bhattacharjee, Ujjayan Majumdar, William S. Dernell, Amit Bandyopadhyay, and Susmita Bose*

W. M. Keck Biomedical Materials Research Laboratory

School of Mechanical and Materials Engineering

Washington State University, Pullman, Washington 99164, USA.

*Corresponding author email: sbose@wsu.edu

Supplementary information

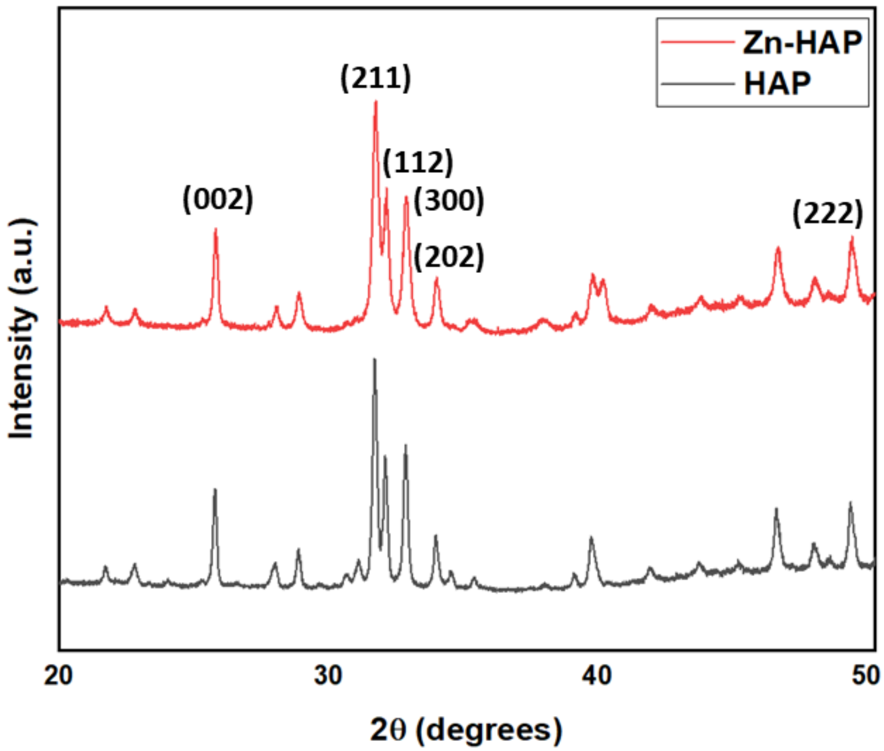

Fig. S1. XRD of HAP and Zn-HAP shows no undesirable phase formation because of doping and plasma-sprayed coating. The generated peaks match well with hydroxyapatite (JCPDS # 09-0432).
